# Structural Dynamics of OATP1A2 in Mediating Paclitaxel Transport Mechanism in Breast Cancer

**DOI:** 10.7150/ntno.103095

**Published:** 2025-02-03

**Authors:** Rohit Kumar, Garima Singh, Yusuf Akhter, Gaurav Kaithwas, Ashish Kumar Agrawal, Sanjay Singh

**Affiliations:** 1 Department of Pharmaceutical Sciences, School of Pharmaceutical Sciences, Babasaheb Bhimrao Ambedkar University (A Central University), Vidya Vihar, Raebareli Road, Lucknow- 226025, Uttar Pradesh, India.; 2 Department of Biotechnology, Babasaheb Bhimrao Ambedkar University (A Central University), Vidya Vihar, Raebareli Road, Lucknow- 226025, Uttar Pradesh, India.; 3 Department of Pharmaceutical Engineering and Technology, Indian Institute of Technology (BHU), Varanasi- 221005, Uttar Pradesh, India.; 4 Dr. Shakuntala Misra National Rehabilitation University, Mohaan Road, Lucknow- 226017, Uttar Pradesh, India.

**Keywords:** OATP1A2, Protein Homology Modeling, Protein-ligand Docking, Molecular Dynamics, Membrane Transporter, Paclitaxel.

## Abstract

Breast cancer remains a significant global health challenge, with drug resistance and poor bioavailability of chemotherapeutic agents like paclitaxel (PTX) presenting obstacles to effective treatment. This study investigates the potential role of the Solute Carrier Organic Anion Transporter Polypeptide 1A2 (OATP1A2) in PTX transport using computational approaches.

We employed computational modeling, molecular docking, and molecular dynamics (MD) simulations to elucidate the structural dynamics of OATP1A2 and its interaction with PTX. The OATP1A2 structure was modeled using Phyre2, validated, and refined. Molecular docking revealed significant PTX interactions within the predicted binding site, with a binding affinity of -10.4 kcal/mol and initial hydrogen bonding with Arg^656^ and Gly^560^ and hydrophobic interaction with atGlu^66^, Phe^65^, Asn^41^, Ala^203^, Ile^204^, Phe^329^, Phe^332^, Ile^336^, Pro^207^, Ser^337^, Asn^334^. Contrary to our initial hypothesis of inward drug movement, MD simulation over 500 ns revealed an unexpected outward movement of PTX. The ligand shifted approximately 5.4 Å towards the extracellular side from its initial binding position. This observation suggests a more complex transport mechanism than initially anticipated. The protein-ligand complex exhibited stability throughout the simulation, with notable conformational changes. Our findings highlight the complex nature of OATP1A2-mediated transport and its potential limitations for PTX delivery. These results accentuate the complexity of transporter-mediated drug delivery and may inform future strategies for improving chemotherapeutic efficacy in breast cancer treatment.

## Introduction

Cancer is a disease characterized by the disruption of normal cell progression, leading to uncontrolled cell division due to the failure of apoptosis (programmed cell death). Over time, this unregulated growth can result in the formation of a solid tumor 1. Breast cancer is the most common solid tumor in women globally after lung cancer and is the second leading cause of cancer-related death in women. In India, breast cancer is diagnosed every four mins, with an incidence rate of 10.4% among all malignancies in Indian women 2.

The solute carriers (SLCO) gene family encodes the largest family of membrane transport proteins known as organic anion transporter polypeptides (OATPs). These transporters play a crucial role in drug transportation in breast carcinomas. OATPs are Na^+^ independent transporters present in humans (OATPs) and animals (Oatps), responsible for transporting various amphipathic, endogenous, and exogenous organic compounds 3. OATPs consist of 643-722 amino acids and contain 12 transmembrane (TM) helices separated by six external and five intracellular loops, with both the C-terminal and N-terminal ends of the polypeptide chain located on the cytoplasmic side of cells. Computational research has suggested a common structure for OATPs, indicating an evolutionary link within the Major Facilitator Superfamily (MSF) 4**.** In humans, rats, and mice; 36 OATP/SLCO superfamily members have been discovered so far. These members are divided into six families (OATP1-OATP6) and 13 subfamilies 5**.** Transporters of the OATP family mediate cellular uptake of a variety of clinically important drugs, including various statins, angiotensin II receptor antagonists, such as olmesartan and valsartan, and the endothelin receptor antagonist bosentan. OATP family transporters are recognised to have major impacts on the pharmacokinetic, safety and efficacy profiles of these drugs 6. Human OATPs are key uptake transporters found in a variety of tissues including the liver, kidney, placenta, and blood-brain barrier 7**.** OATPs are mostly found in hepatocytes, but other studies have shown their presence in cancer tissues of the liver, colon, breast, pancreas, and prostate cancer cells 8. Studies also suggested that these are antiporter or symporter in nature, although their exact nature is unknown 9. Among the OATP family members, OATP1A2 has been significantly expressed in human breast cancer, as confirmed by quantitative RT-PCR. The epithelia of invasive ductal carcinomas of breast tissue express both OATP1A2 and OATP2B1 show higher expression in the breast cancer patient cancer cells 10-12. Paclitaxel (PTX), discovered over more than 12, 000 natural chemicals with anticancer potential, is a poorly water-soluble and highly lipophobic substance 13. PTX is primarily transported by OATPs in breast cancer membranes and has less or minimal diffusion process. The OATP protein transporter has shown a high affinity for PTX, or taxol 14. PTX was shown to interact appropriately with dipalmitoyl phosphatidylcholine (DPPC), lowering their interaction energy 15. Multidrug resistance (MDR) in cancer therapy is mostly caused by cancer cells remodeling their plasma membrane to encourage proliferation, avoid apoptosis, and resist anticancer agents 29. Another most frequent evident of cells that are resistant to many drugs is the over-expression of ABC transporters, including P-glycoprotein (Pgp). 28. Given the above, the present study has been undertaken to study the role of OATP1A2 membrane transporter for PTX uptake and evaluate its efficiency in terms of membrane permeability and pharmacokinetics of PTX.

## Methodology

### Retrieval of sequence dataset

**Figure 1:**
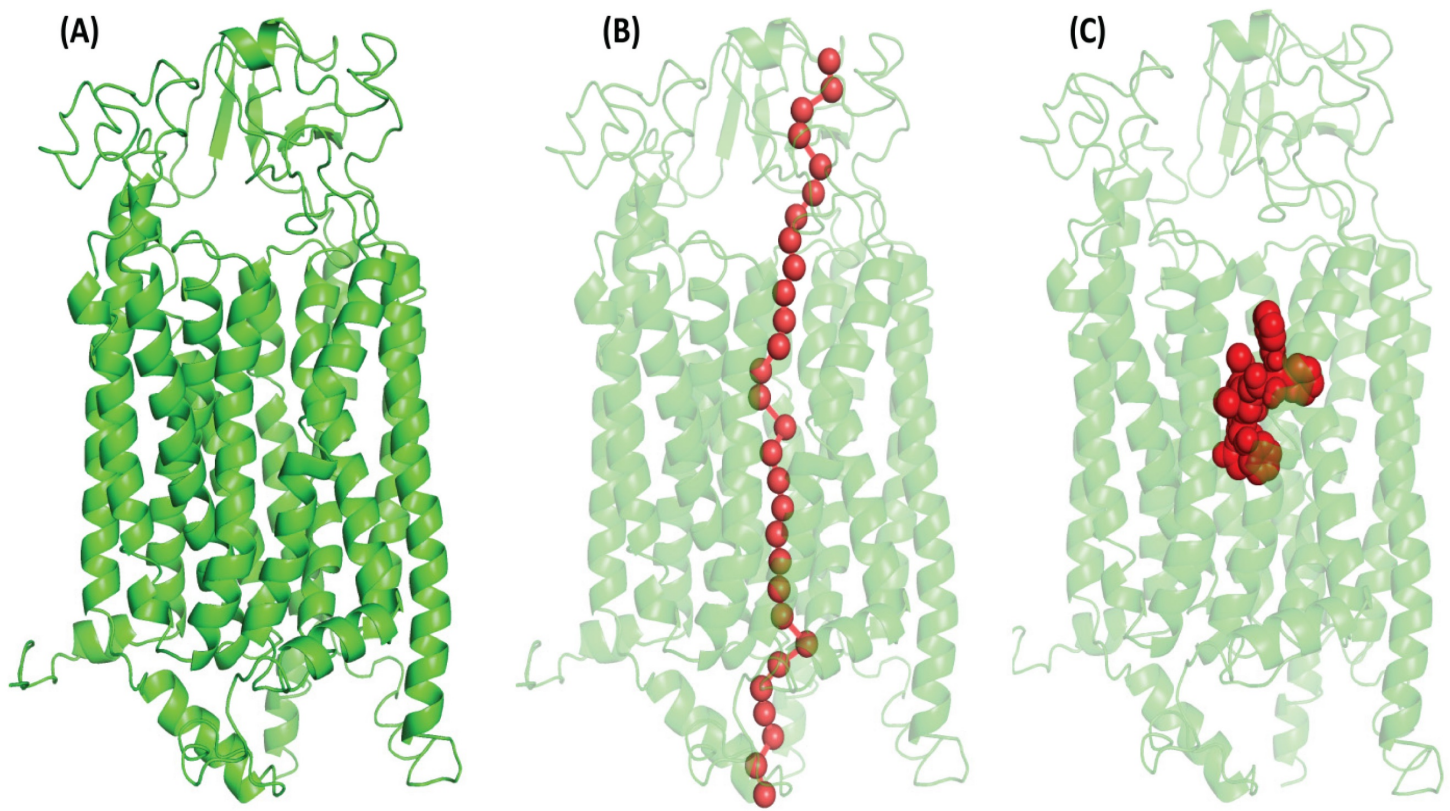
(A) Illustrate the OATP1A2 protein's 3-dimensional structure, generated through structural homology modeling by the Phyre2 web server which has inward-open conformation with two N- TAD and C-TAD domains constructed from TM helices. (B) PoreWalker analyses showed the putative tunnel with overlapping binding site residues that passes through the protein's core cavity (C) Red doted sphere shape (in middle) denoted the protein's binding pocket and highlights the amino acid residues actively involved (Lys^33^, Arg^168^, Glu^173,^ and Arg^556^) in the interaction with OATP1A2.

The primary sequences of OATP1A2 were obtained from the UniProt online web server (https://www.uniprot.org) of Humans (*Homo sapiens*) in FASTA format (UniProt id P46721). UniProt is the online server that provides a complete protein dataset and collects all protein sequence information 16. The complete experimental process of the study has been represented in the form of a flow diagram (**S. Figure 1**).

### Structural homology modeling

Online computational tools like Phyre2, I-Tasser, and the Swiss model were used to build the tertiary structure of proteins. Briefly, the sequences collected from the UniProt web server were submitted as a query to Phyre2 (accessed on 7.03.2022, 8:26 PM), I-Tasser (Accessed on 12.01.2022, 6:06 PM), and Swiss model (accessed on 15.03.2022, 3:00 PM) respectively. These programs are based on the fold recognition theory and methods to build the protein structure. It uses templates of homologous proteins to construct 3-dimensional protein structures through multiple stages. The algorithm aligned many templates to create the final structure of the proteins 17.

### Validation and energy minimization

Protein structure refinement tools KoBaMIN and 3Drefine were used to improve the quality of the modeled protein's 3-dimensional structure. Both tools have an online platform for atomic-level energy minimization-based protein structure improvement and hydrogen-bond network optimization using knowledge-based force fields 18. The PROCHECK tool (Accessed on 17.06 2022, 12:13 PM) was used to evaluate the Ramachandran plot for the protein structure model's secondary structural characteristics and stereochemistry 19.

### Protein topology

The annotation of the protein was predicted by using the algorithm Deep HMMMTv 2.0 (transmembrane protein topology hidden Markov model) (https://services.healthtech.dtu.dk/services/TMHMM-2.0/). Protein topology prediction aims to determine the spatial arrangement of secondary structures (like alpha-helices and beta-strands) within a protein sequence.

### Binding pocket and tunnel detection

To the binding location firstly, we searched the literature for critical residues involved in the substrate interaction, and then we submitted the structure as a query to the ligand-binding prediction computational tools COACH (Accessed on 22.11.2022, 3:02 PM) and 3-D-LigandSite (Accessed on 23.11.2022, 11:32 PM) 20. The PoreWalker computation tool (Accessed on 27.09.2022, 6:32 PM) found the channel for the transportation of ligands and residues involved in transportation. It uses heuristic and iterative techniques to detect and identify a TM protein canal in a 3D protein structure. It locates and optimizes the pore axis's centre, finds the optimal cavity, and analyses pore characteristics such as shape, size, reliability, pore-lining atoms, and residues 21.

### Ligand retrieval

The 3D structure of the PTX was obtained from ChemSpider online database (Id 10368587) (http://www.chemspider.com/) in mol format.

### Molecular Docking

The AutoDock Tools 1.5.7 and AutoDock Vina packages were used to perform the molecular docking analysis 22. Protein was given a basic structure by adding Kollman charges and polar hydrogen. The protein and ligand (PTX) were then converted to PDBQT format. The grid box was placed after the x, y, and z-axis coordinates of the receptor molecule were 60.832, 67.402 and 57.155 with sizes 60, 68, and 72. Grid coordinates were established, 4 Å spacing to encompass all residues involved in the substrate binding pocket, to optimize drug binding at the expected binding pocket. Before moving to the structural investigation of the complexes, the ligand was re-docked using known locations. Following docking, the LigPlot^+^ program was used to generate a variety of ligand 2D states depending on interactions and binding affinities 19.

### Molecular dynamic (MD) simulation

The Desmond was utilized to perform the MD simulation, applying the OPLS3e force field. The systems were produced using the water solvation model (12796 molecules) and inserted into a DPPC (dipalmitoyl phosphatidylcholine) bilayer membrane that had been pre-equilibrated at a temperature of 300.0 K with a total -7 Clˉ counterions were used. The systems were created with an orthorhombic box and a buffer distance of 10 Å, utilizing an SPS environment. The membrane was positioned at a right angle to the central pore of the protein-ligand complex, aligning with the shape of the helices. The prepared systems were subjected to a simulation of at 500 ns. The simulation was performed using 3 copies, each utilizing different seeds for the velocities. The simulation was carried out in the NPγT ensemble at a temperature of 300 Kelvin. The simulations utilized a Nose-Hoover chain thermostat to regulate temperature, while the pressure was kept constant at 1.01 bar using a Martyna-Tobias-Klein barostat. The default relaxation technique was implemented using the RESPA integrator with a time step of 2 fs for bonded atom pairs. Non-bonded interactions were handled separately for near (2 fs) and far (6 fs) atom pairings. A 9 Å cut-off radius was established for short-range coulombic interactions. The Simulation Event Analysis pipeline, developed in Maestro 2020.2 (Schrödinger LCC) 23, was used to estimate atomic interactions and distances. MD trajectories were viewed, and graphics were generated using PyMol 5.7 (Schrödinger LCC, New York, NY, USA) 24. The simulation results were analyzed using Maestro 2020.2 (Schrödinger LCC). Trajectories were extracted at different time intervals to calculate various parameters for the complex. These parameters include Root Mean Square Deviation (RMSD), Root Mean Square Fluctuations (RMSF), hydrogen bond interactions, radius of gyration (Rg), and distance between core atoms.

## Results

### Phyre2 generated model of OATP1A2 demonstrated the best quality among various modeling methods

**Figure 2:**
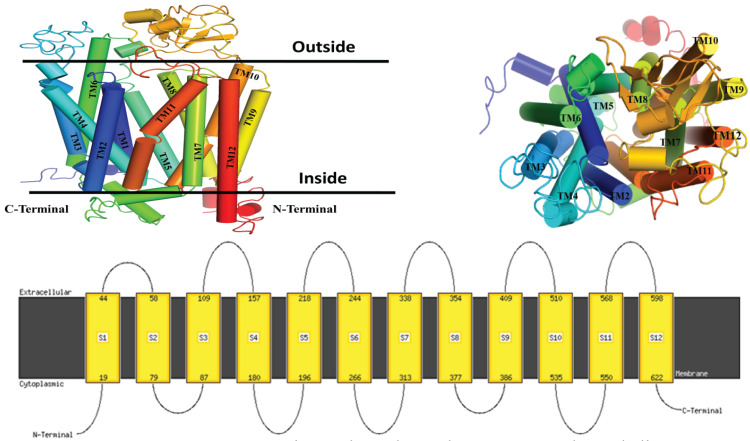
Illustrate the topology of a 3-dimensional protein structure, generated through structural homology modeling, which comprises the 12 helices, 6 extracellular loops and 5 intracellular with an inward-open conformation with two N- TAD and C-TAD domains both are cytoplasmic site.

The Catspermasome transporter (PDB ID-7eeb) was shown to have 100% confidence of the amino acid sequence in homology similarity with OATP1A2 (**S. Figure 2**). It is commonly assumed that proteins have a similar structured Catspermasome transporter protein. Among the web servers, the model developed by Phyre2 showed superior query coverage, confidence levels, and overall model quality, and the innermost open model was chosen for further study (**Figure 1A**).

### Validated OATP1A2 model reveals twelve-helices structure with cytoplasmic terminal

**Figure 3:**
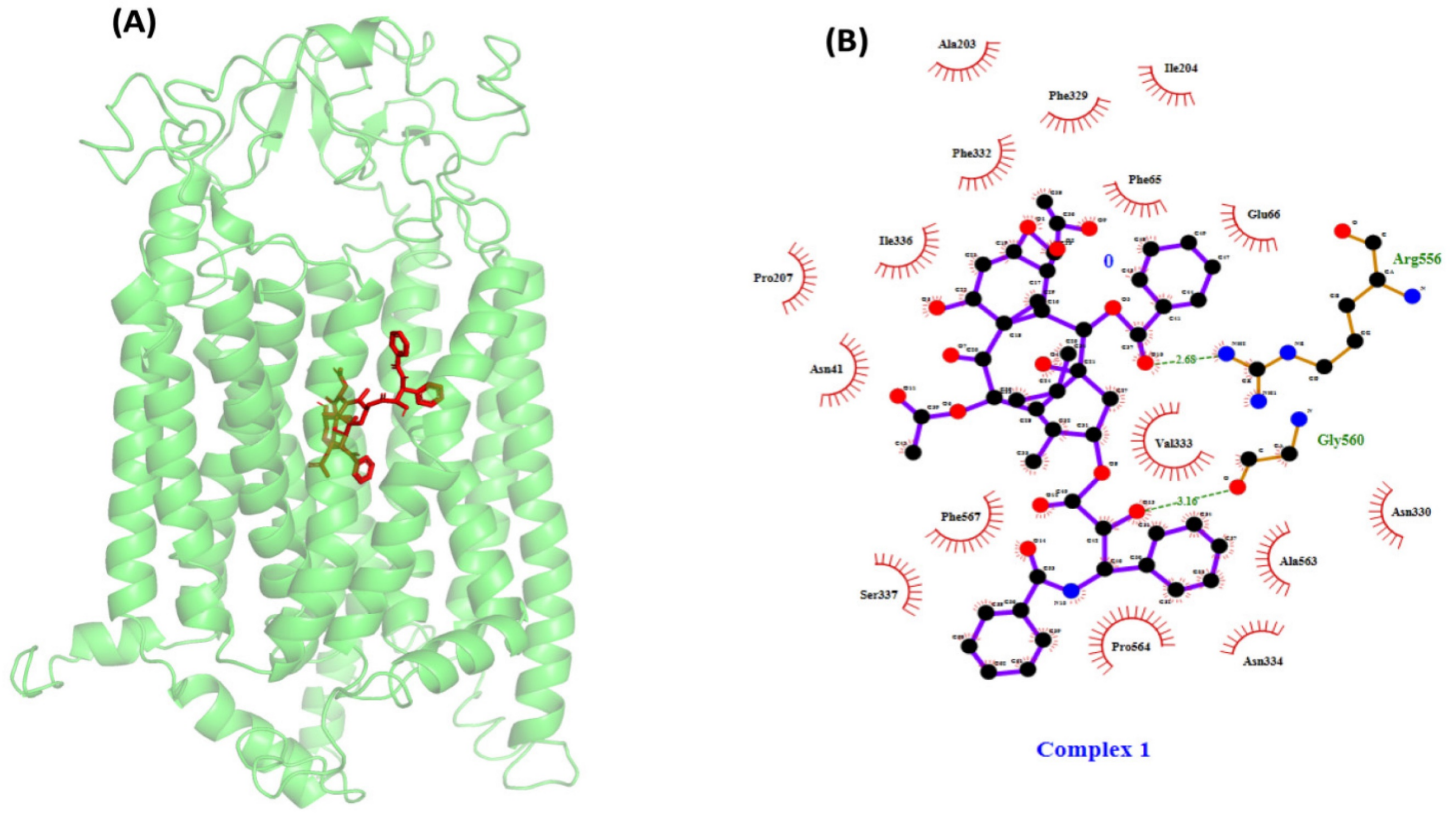
(A) Illustrates the protein-ligand docking complex performed by AutoDock Vina package1.5.7. The protein showed in green color carton structure and the red stick present in the center of the protein indicates the paclitaxel. (B) Shows the protein-ligand interaction in 2D structure, the ligand showed hydrogen bonding with Arg^656^ and Gly^560^ visualized by Ligplot and many hydrophic bond likeGlu^66^, Phe^65^, Asn^41^, Ala^203^, Ile^204^, Phe^329^, Phe^332^, Ile^336^, Pro^207^, Ser^337^, and Asn^334^.

Ramachandran plot was used to assess the quality of protein. It showed 87.5% atomic residue present in the favoured region. 11% showed an additional favoured region and 0.6% present is the only residue in the disallowed region which means the modeled protein by Phyre2 was suitable for further experimentation **(S. Figure 3)**. The topology of the protein revealed that the protein has twelve TM helices. The Six loops are situated at the extracellular and five loops are intracellular. The assignments for these TM helices were: H1 (Met^19^ to Thr^44^), H2 (Glu^58^ to Lys^79^), H3 (Lys^87^ to Arg^109^), H4 (Thr^157^ to His^180^), H5 (Arg^196^ to Ile^208^), H6 (Arg^244^ to Leu^266^), H7 (Leu^313^ to Lys^338^), H8 (Met^354^ to Phe^377^), H9 (Phe^386^ to Leu^409^), H10 (Phe^510^ to Ser^535^), H11 (Lys^550^ to Thr^568^), and H12 (Ser^598^ to Ser^622^). Both the N-TAD and C-TAD were found to be oriented in the cytoplasmic direction of the structure **(Figure 2)**.

### PoreWalker detected a central tunnel with hydrophobic residues lining from extracellular to intracellular Side

The PoreWalker algorithm created a single central tunnel for tunnel identification, which crossed the protein at the N-C domain contact and went from the extracellular to the intracellular side. Most of the amino acids that made up the pore residues were alanine, threonine, valine, leucine, serine, glycine, etc. The transport channel predicted by PoreWalker overlapped with the amino acid residues in the substrate-binding pocket (**Figure 1B**).

### Docking studies revealed that PTX binds to a conventional Major Facilitator Superfamily (MFS)-like the pocket in OATP1A2

Typically, MFS proteins are found to have a single substrate-binding pocket midway along the membrane. PTX was docked using a conventional binding pocket that was identified from literature, COACH, 3-DLigandSite, and the one that was determined based on a pore walker. The binding pocket represented amino acid residues Lys^33^, Arg^168^, Glu^173,^ and Arg^556^ 25 **(Figure 1C)**.

### PTX-OATP1A2 docking exhibited strong binding through hydrogen and hydrophobic interactions

Following the identification of the likely physiologically significant binding pocket, a docking study was conducted to investigate the interaction between PTX and the OATP1A2 protein at an anticipated binding location. The noted binding affinity of PTX was observed at -10.4 kcal/mol. In addition, the ligand first formed hydrogen bonds with Arg^656^ and Gly^560^. The remaining residues exhibited hydrophobic interactions with ligand, with major interactions at Glu^66^, Phe^65^, Asn^41^, Ala^203^, Ile^204^, Phe^329^, Phe^332^, Ile^336^, Pro^207^, Ser^337^, and Asn^334^ **(Figure 3)**.

### MD simulation showed PTX movement and OATP1A2 conformational changes in the membrane environment

**Figure 4:**
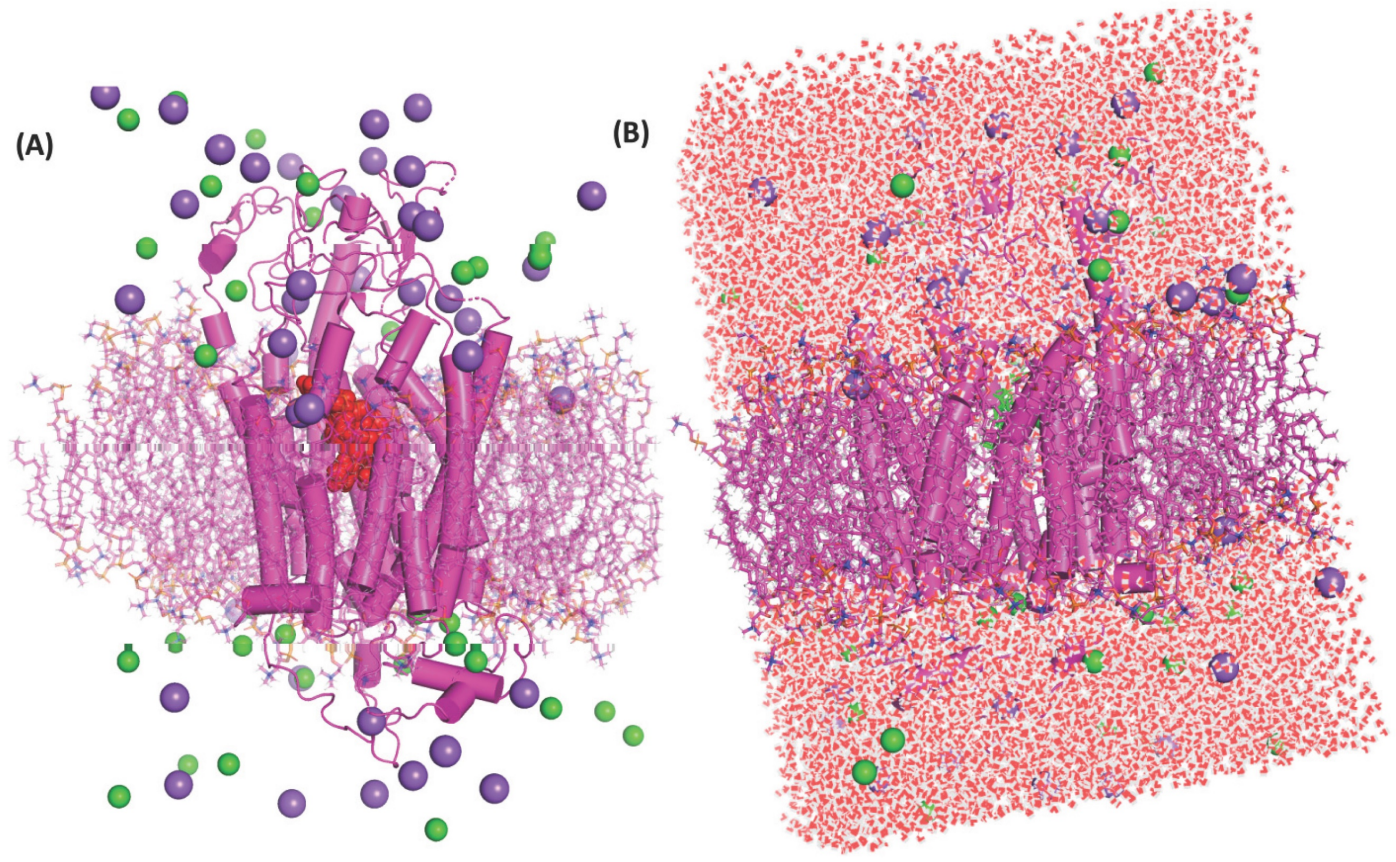
(A) Protein-ligand complex inserted to the DPPC's lipid bilayer, protein showed as the cylindrical shape in magenta colour and ligand in red dotted spherical into the centre of protein this protein-ligand complex were inserted into a magenta colour stick which is DPPC lipids bilayer. Cl^-^ ions denoted spherical shape by green colour and magenta colour dotted spherical shape indicates the Na^+^ ions, green stick denoted the DPPC bilayer membrane. (B) The system-A architecture is shown in full in the figure, and water is passing through the protein's core chamber. Here, red and white dots represent the molecules of water, Cl^-^ ions are denoted by green colour and magenta colour dotted indicates the Na^+^ ions, green stick denotes the DPPC bilayer membrane.

**Figure 5:**
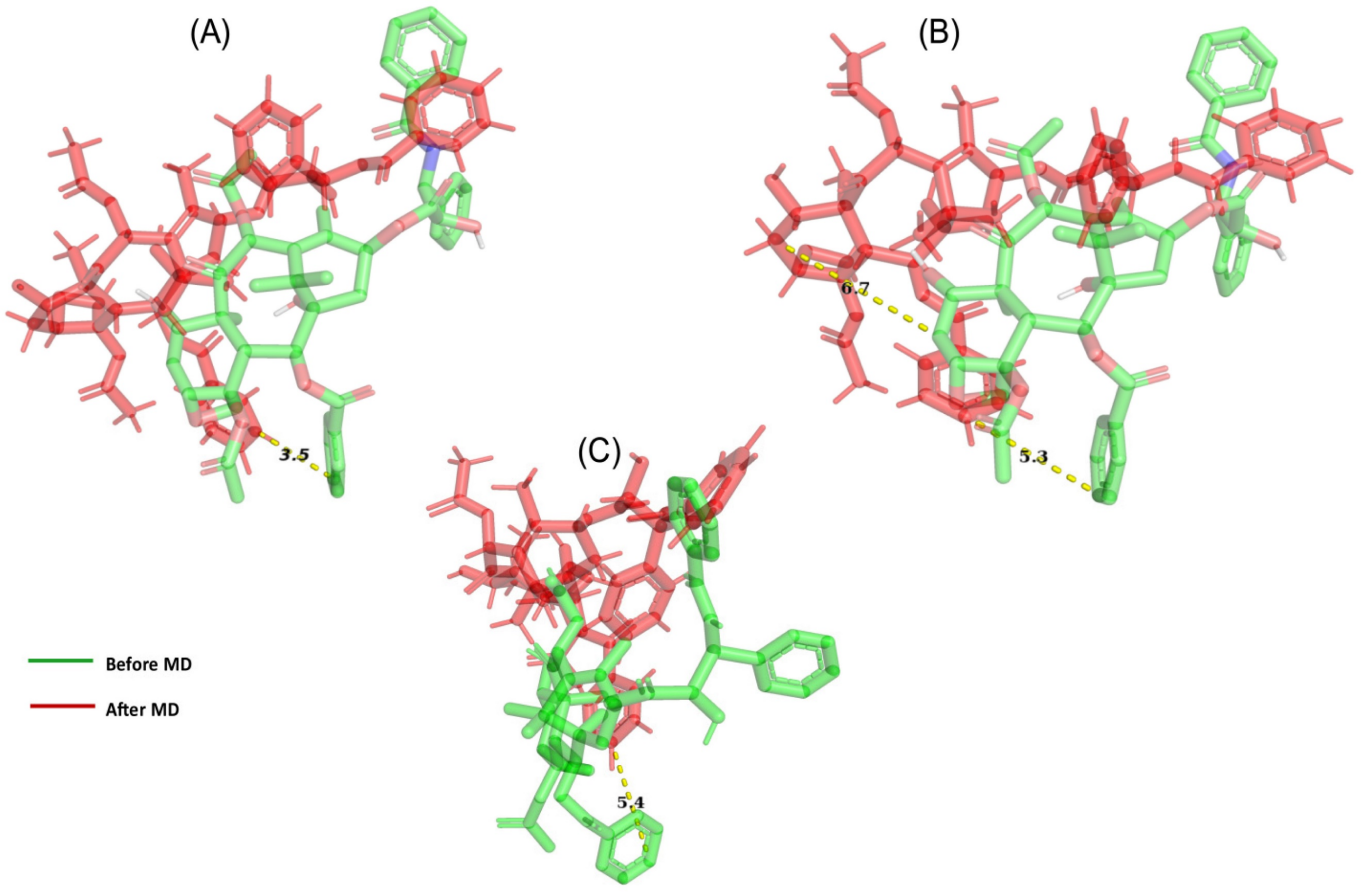
Demonstrate the movement of ligand throughout the protein (A) after 50 ns of simulation, a net movement to the initial location was 3.5 Å seen with the OATP1A2 protein in complex with OATP1A2. (B) After 200 ns we observed 5.3 Å movements toward upward and right sides from its initial site. (C) After 500 ns, we observed a major movement of 5.4 Å toward upward.

**Figure 6:**
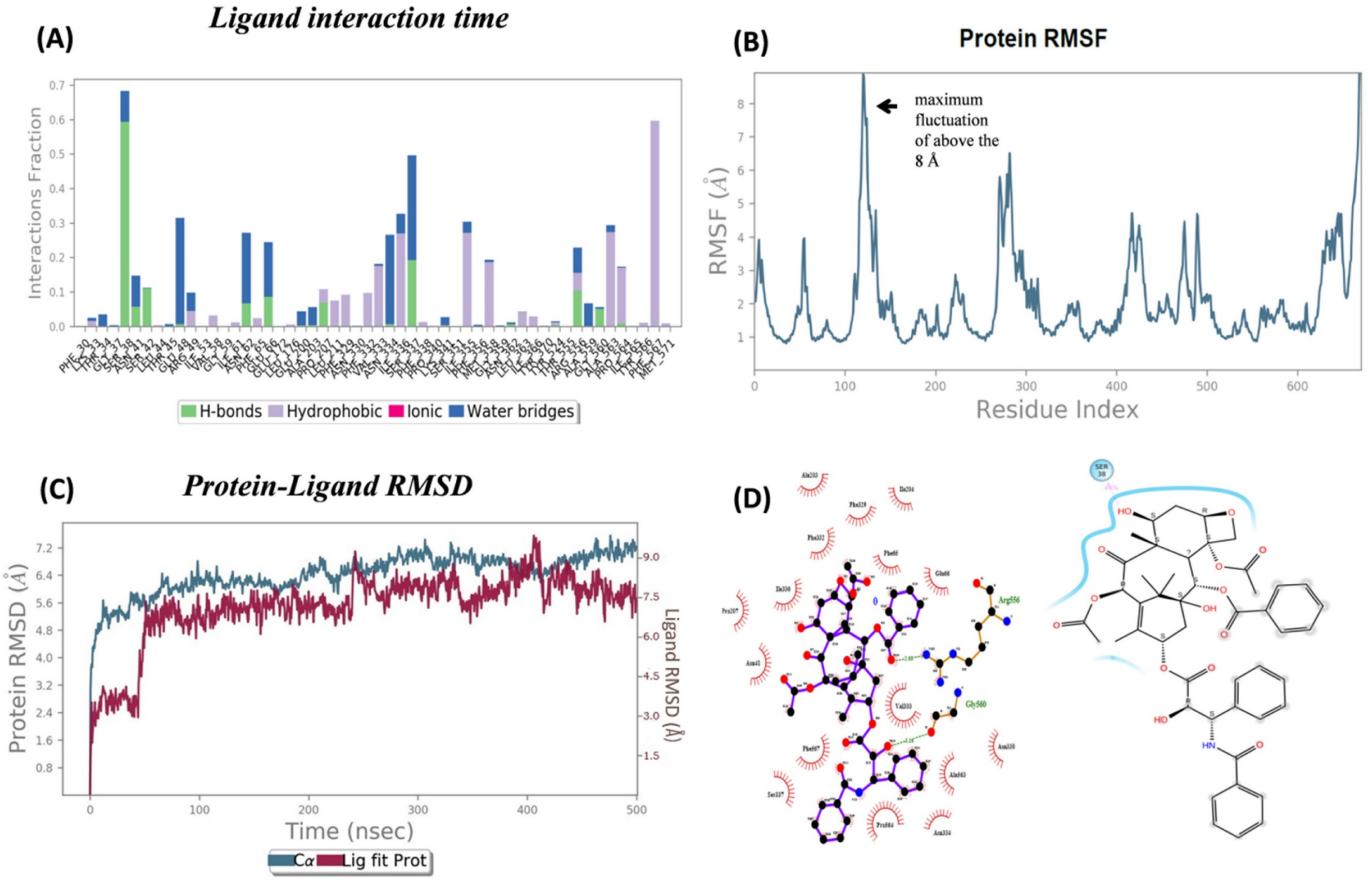
Ligand-protein analysis and conformation changes during MD simulation. (A) Chart shows the type of interaction fraction time (Shown in different colors for each interaction) of protein containing amino acid during the 500 ns MD simulations. However, As the MD simulation began and gradually increased, the hydrogen bond decreased. The highest contact times are shown by residues such as Phe469, Ile204, and Arg656, which suggest important roles. **(B)** OATP1A2 RMSF calculations illustrate the flexibility during MD simulation, A connecting amino acids from 100-150 in OATP1A2 protein revealed the highest fluctuation above the 8 Å. This suggests the extracellular loop is important for ligand transportation **(C)** The RMSD values for the protein-ligand complex were shown in the figure during 500ns MD simulation. The analysis revealed stability throughout the simulation period, with the protein stabilized around6 Å and ligand 1.5 Å, followed by RMSD values remained consistent. After 100ns figure represented the structural integrity and reliability of the protein-ligand interactions over the MD simulation.** (D)** The left side: the figure shows the types of interaction before MD simulation (Docking protein-ligand complex). The hydrogen bond with amino acid Arg^656^ and Gly^560^was observed and the other is hydrophobic interaction. The right side: After MD which shows only hydrophobic interaction over 500ns. Which was reflects the changes in the interaction bond after 500 ns.

A docked protein-ligand complex was placed into the DPPC lipid membrane to mimic physiological conditions** (Figure 4A & 4B)**. During the MD simulation, we observed the ligand dislocated from its initial location or binding site. So far as movement was cornered the ligand slightly moved toward the upper at around 200 ns (5.3 Å when assuming C49 as a centre atom) **(Figure 5B)**. (MD at 500 ns last frame) the ligand was moved extracellular (5.4 Å) as well as the left side and we also observed the ligand when compared with the initial binding position **(Figure 5C)**. Initially, the ligand formed two hydrogen bonds and several hydrophobic interactions within the binding pocket. However, once the 500 ns simulation was completed, it was revealed that the ligand had only hydrophobic interactions since they had travelled up and away from the first binding site into the membrane's periplasmic region** (Figure 6D)**. The results indicate that PTX has the property to show resistance by receptors.

The most notable variations were seen in the amino acid residues that were most abundant in the loop linking the complex at the extracellular site. RMSF calculations were analyzing the PTX-OATP1A2 complex. The transporter protein exhibited a significant conformational shift, between the N-TAD and C-TAD domains in a connecting loop showing the highest fluctuation of almost 8 Å in the PTX-OATP1A2 protein complex **(Figure 6B)**. However, fluctuation peaks of different sizes were also seen all over the protein, suggesting that most of the residues are involved in the activity of the protein as a transporter. RMSD values of 500 ns trajectories were evaluated for OATP1A2 backbone atoms and protein-ligand complex and they revealed stability throughout the simulation (**Figure 6C**).

**Figure 7:**
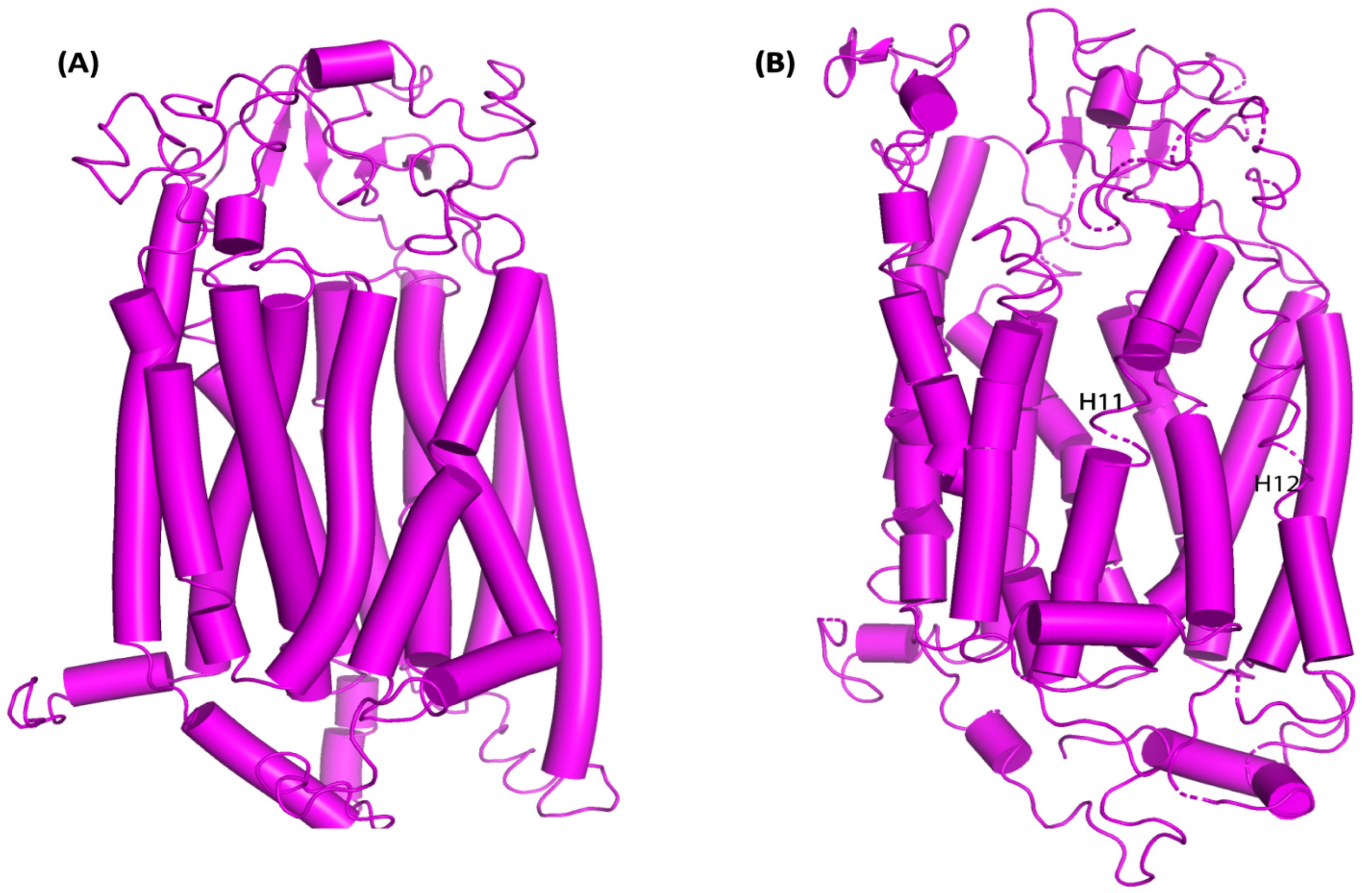
OATP1A2 transporter conformational changes both before and after MD simulations. (A) OATP1A2's structure before the MD simulation (0 ns), displaying the TM helices and loops' original conformation. Particularly between TM helices H11 and H12, the loops show a highly compact shape with the helices closely packed together. (B) OATP1A2's structure following 500 ns of MD simulation. There is some rearrangement of the TM helices, especially in the vicinity of H11 and H12, where the loops are more flexible and exhibit conformational change. These motions point to a shift in the direction of an outward-open state, which promotes ligand transport.

When examining how the structure of proteins adjusts during MD simulations. Inside the protein complex, there was also a noticeable change in loops between H11 and H12 into parallel beta-sheets. After 80 ns of simulation, the protein structure changed from an inward-open state to an intermediate occluded state, suggesting the possible role of these protein domains in the expulsion of the drug **(Figure 7A & 7B)**.

## Discussion

PTX is an anti-cancer drug widely used for lung cancer, prostate, and breast cancer. It showed resistance and low membrane permeability. It may occur due to its large structure and high molecular weight. Our hypothesis confirms the mechanism of PTX for the transportation inside membrane cells by the OATP1A2 membrane protein transporter which is significantly expressed in breast cancer patients 12. The OATP1A2 Na^+^ independent polypeptide transporter and its mechanism are still unknown, but few studies reported that OATP1A2 followed a rocker-switch mechanism 26, 4.

The PDB database did not contain the 3D- structure of the protein. The fold recognition technique was utilized to construct a 3-dimensional model of the protein's structure. The model that was built had query coverage of 89% and a confidence level of 100%, providing evidence that the query and template sequences were homologous. The sequence similarities between the 3-dimensional structure and the crystal structure with the Catspermasome transporter (PDB ID-7eeb) were considered to serve as a template for Phyre2 to utilize building the 3-dimensional protein structure **(Supplementary Fig. 1)**. The protein consists of N-TAD and C -TAD domains that combine to create a unique central cavity located at the interface between the two domains. One domain possesses six TM helices located extracellular, whilst the other domain contains five helices situated intracellular. The C-TED and N-TED ends approached the side of the cytoplasm. The modeled protein exhibited an inward-open conformation, similar to other drug efflux pumps. This conformation allows the protein to take up pharmaceuticals from the cytoplasm and export them outside the cell. The protein undergoes a transitional structural change to the intermediate-occluded state and subsequently to the outward-open state during this process. However, OATP1A2 has a higher percentage of outside open state. It may indicate the protein uptake the drug from extracellular and move it inside the cell.

Before energy minimization, the structural validity of the OATP1A2 protein's 3-dimensional model was verified. Energy minimization is a crucial process that aims to decrease the potential energy of the entire system to achieve maximum structural and conformational stability. It functions by addressing irregular protein shapes to alleviate internal limitations and construct more robust structures with accurate stereochemistry. Consequently, this stage was important in generating an improved, top-notch 3-dimensional structure. In addition, the dependability of the energy minimization process was assessed via presenting both the energy-minimized and refined structure of OATP1A2 protein to structural validation using PROCHECK to examine the Ramachandran plot. The results indicate the outstanding stability of the energy-minimized protein structure, with a greater proportion of residues located in the preferred and permissible areas. We identified a core transport tunnel that connects the cytoplasmic and periplasmic ends of the OATP1A2 protein, indicating the molecule's possible path during movement. As many studies reported, the OATPs or OATP1A2 protein has a binding pocket in the centre of the protein 25, 26. We also predicted ligand-binding sites using COACH and 3-D-LigandSite for protein, and the results were identical to those published by Arun Kumar Tonduru *et al.* 27 **(Figure 1C).**

Our computational modeling and molecular docking studies demonstrated strong initial binding of PTX to OATP1A2, with a binding affinity of -10.4 kcal/mol and hydrogen bond interactions with Arg^656^ and Gly^560^. These findings suggested a potential role for OATP1A2 in PTX transport. RMSD of the protein backbone atoms for OATP1A2 interact with ligand revealed very few deviations, indicating that the complex stayed intact and, therefore, stable throughout the 500 ns simulation period without acquiring any thermodynamically unfavourable conformations for systems (**Figure 6C**). RMSF calculation was done to quantify each peak of an amino acid residue. The OATP1A2 protein loop connecting its N and C -TAD domains had the most noticeable disordered peak during analysis when compared to helices (**Figure 6B**); the reason for these variations could be that MFS transporters only operate in one conformation at a time through a rocker switch mechanism. In this instance, the OATP1A2 protein was first confirmed to be inwardly open; it then absorbed the drug from the cytoplasm, undergoing conformational changes that resulted in an intermediate occluded state and, finally, an outwardly open state. As hydrogen bonds are important interactions between biomolecules involved in numerous biological processes, we included calculations of hydrogen bonds in our analysis. H-bonds between OATP1A2 protein-ligand complex were figured out. Initially, two hydrogen bond contacts were first seen in the complex; however, following MD, they began to decrease, and hydrophobic interactions increased (**Figure 6A**). The collected data can accurately explain the cause of this declining H-bond interaction. As the simulation duration increased, the ligand molecules drifted away from the original binding site and toward the periplasmic side to efflux. As a result, there were more protein-ligand interactions at the binding site, and these associations tended to decrease as soon as the ligand departed.

Additionally, we assessed the system's compactness during the simulation by measuring the radius of gyration (Rg). If protein is firmly folded, Rg value would remain comparatively constant. However, if protein unfolds, the Rg value would change eventually. During the simulation, Rg value for the complex remained consistently compressed, indicating that the protein maintained its overall structural integrity. Water molecules were subsequently introduced into the systems and caused them to become solvated. The trajectory analysis revealed that the water molecules followed a path within the transport canal, transferring the lipid bilayer system's top to lower leaflets. MD simulations facilitated the analysis of structural modifications in the OATP1A2 protein after PTX translocation across systems. When presented as a graph, the protein backbone RMSD for protein-bound PTX shows negligible variations. It clearly shows that the complex during the 500 ns simulation time was intact and stable, without gaining any thermodynamically unfavorable conformations for the systems.

We observed outward movement towards the extracellular space of PTX in comparison to anticipated inward movement. The above findings are not in line with our hypothesis that OATP1A2 would facilitate the uptake of PTX. This unexpected result could be attributed to several factors. Firstly, OATP1A2 may have a more intricate transport cycle than initially assumed, possibly involving multiple steps or conformational changes. Additionally, the 500 ns time frame might not have captured the complete transport cycle, or the initial setup may have influenced the observed movement. Furthermore, OATP1A2 might possess both influx and efflux capabilities under certain conditions.

Sun and colleagues reported that PTX showed resistance and it's a major problem in chemotherapy of cancer and also highlighted the challenges of transported-based PTX delivery 28. The slow or potentially outward movement observed in our simulations suggests that relying solely on OATP1A2-mediated transport may not be the most efficient method for PTX delivery in breast cancer treatment. Interestingly, our parallel investigations into nanoformulation-based delivery of PTX yielded promising results. This outcome aligns with our initial hypothesis that nanoformulation could provide a more effective mode of drug delivery. The contrasting results between the transporter-based and nanoformulation approaches underscore the importance of exploring multiple strategies in drug delivery research 29. While OATP1A2 may play a role in PTX transport, our findings suggest that its contribution might be limited or more complex than initially anticipated. On the other hand, nanoformulation appears to offer significant advantages, potentially by enhancing drug solubility and bioavailability, bypassing efflux mechanisms that might affect transporter-mediated uptake, and facilitating more rapid and efficient cellular uptake through alternative pathways.

These insights have important implications for the development of effective PTX delivery systems in breast cancer treatment. While further research is needed to fully understand the intricacies of OATP1A2-mediated transport, our results strongly support the pursuit of nanoformulation strategies as a promising avenue for improving PTX delivery.

## Conclusion

The analysis of OATP1A2-mediated transport revealed unexpected dynamics, with PTX showing a tendency for outward movement rather than the anticipated inward transport. This finding highlights the complexity of transporter-mediated drug delivery. It also suggests limitations in relying solely on OATP1A2 for effective PTX uptake. The contrasting results between transporter-mediated and nanoformulation-based approaches emphasize the importance of exploring multiple strategies in drug delivery research. While OATP1A2 may play a role in PTX transport, its contribution appears to be more complex than initially anticipated. The authors would like to conclude that the delivery through transporter-mediated mechanisms poses diversified challenges and utilizing a formulation-based approach may be a useful strategy to meet the challenges. Taking insight from both strategies, we can work towards developing more effective and targeted therapies, ultimately aiming to improve patient outcomes in breast cancer treatment.

## Supplementary Material

Supplementary figures.

## Abbreviations

OATPs: Organic anion transporter polypeptides
3D: 3-Dimensional
MSF: Major Facilitator Superfamily
PTX: Paclitaxel
TAD: Terminal
S.: Supplementary
DPPC: Dipalmitoyl phosphatidylcholine
MD: Molecular Dynamic
RMSD: Root means square Deviation
RMSF: Root means square Fluctuations
Rg: Radius of gyration
