# Supplementary material for: Structural Dynamics of OATP1A2 in Mediating Paclitaxel Transport Mechanism in Breast Cancer
Source: Nanotheranostics. 2025 Feb 3;9(1):52–62. doi: 10.7150/ntno.103095 (PMC11898720; doi:10.7150/ntno.103095)
Supplement: Supplementary file 1 — Supplementary figures. [file ntnov09p0052s1.pdf]

# Supplementary figure 1

| # | Template | Alignment Coverage | 3D Model | Confidence | % i.d. | Template Information |
|---|---|---|---|---|---|---|
| 1 | c7eebL_ | Alignment | 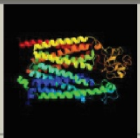 | 100.0 | 22 | **PDB header:**protein transport<br>**Chain:** L: **PDB Molecule:**kazal-like domain-containing protein;<br>**PDBTitle:** structure of the catspermasome<br>**PDB Entry:** PDBe RCSB PDBj |
| 2 | c6rw3D_ | Alignment | 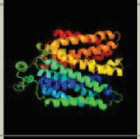 | 99.9 | 18 | **PDB header:**membrane protein<br>**Chain:** D: **PDB Molecule:**hexose transporter 1;<br>**PDBTitle:** the molecular basis for sugar import in malaria parasites.<br>**PDB Entry:** PDBe RCSB PDBj |
| 3 | c7bp3A_ | Alignment | 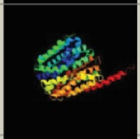 | 99.8 | 19 | **PDB header:**transport protein<br>**Chain:** A: **PDB Molecule:**monocarboxylate transporter 2;<br>**PDBTitle:** cryo-em structure of the human mct2<br>**PDB Entry:** PDBe RCSB PDBj |
| 4 | c6kklA_ | Alignment | 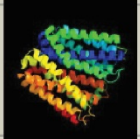 | 99.9 | 20 | **PDB header:**transport protein<br>**Chain:** A: **PDB Molecule:**sugar efflux transporter;<br>**PDBTitle:** crystal structure of drug:proton antiporter-1 (dha1) family sotb, in2 the inward conformation (h115n mutant)<br>**PDB Entry:** PDBe RCSB PDBj |
| 5 | c7pmwA_ | Alignment | 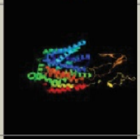 | 99.2 | 14 | **PDB header:**membrane protein<br>**Chain:** A: **PDB Molecule:**solute carrier family 15 member 1;<br>**PDBTitle:** hspept1 bound to ala-phe in the outward facing occluded conformation<br>**PDB Entry:** PDBe RCSB PDBj |
| 6 | c7ckrA_ | Alignment | 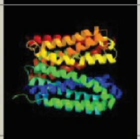 | 99.9 | 19 | **PDB header:**transport protein<br>**Chain:** A: **PDB Molecule:**monocarboxylate transporter 1;<br>**PDBTitle:** cryo-em structure of the human mct1/basigin-2 complex in the presence2 of anti-cancer drug candidate bay-8002 in the outward-open3 conformation.<br>**PDB Entry:** PDBe RCSB PDBj |
| 7 | c6v4dA_ | Alignment | 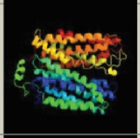 | 99.9 | 17 | **PDB header:**membrane protein<br>**Chain:** A: **PDB Molecule:**vesicular glutamate transporter 2;<br>**PDBTitle:** structure of the rat vesicular glutamate transporter 2 determined by2 single particle cryo-em<br>**PDB Entry:** PDBe RCSB PDBj |
| 8 | c6e8jA_ | Alignment | 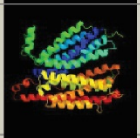 | 99.9 | 16 | **PDB header:**transport protein<br>**Chain:** A: **PDB Molecule:**major facilitator family transporter;<br>**PDBTitle:** crystal structure of a bacterial homolog to human lysosomal2 transporter, spinster, in inward-facing and occupied conformation<br>**PDB Entry:** PDBe RCSB PDBj |
| 9 | c6gs7A_ | Alignment | 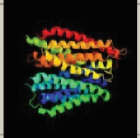 | 99.9 | 16 | **PDB header:**membrane protein<br>**Chain:** A: **PDB Molecule:**dipeptide and tripeptide permease a;<br>**PDBTitle:** crystal structure of peptide transporter dtpa-nanobody in glycine2 buffer<br>**PDB Entry:** PDBe RCSB PDBj |
| 10 | c6g9xB_ | Alignment | 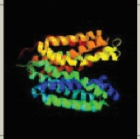 | 99.8 | 18 | **PDB header:**membrane protein<br>**Chain:** B: **PDB Molecule:**major facilitator superfamily mfs_1;<br>**PDBTitle:** crystal structure of a mfs transporter at 2.54 angstroem resolution<br>**PDB Entry:** PDBe RCSB PDBj |
| 11 | c4zp0A_ | Alignment | 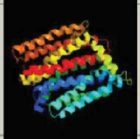 | 99.9 | 18 | **PDB header:**transport protein<br>**Chain:** A: **PDB Molecule:**multidrug transporter mdfa;<br>**PDBTitle:** crystal structure of e. coli multidrug transporter mdfa in complex2 with deoxycholate<br>**PDB Entry:** PDBe RCSB PDBj |

Supplementary Figure 2

PROCHECK

# Ramachandran Plot

saves

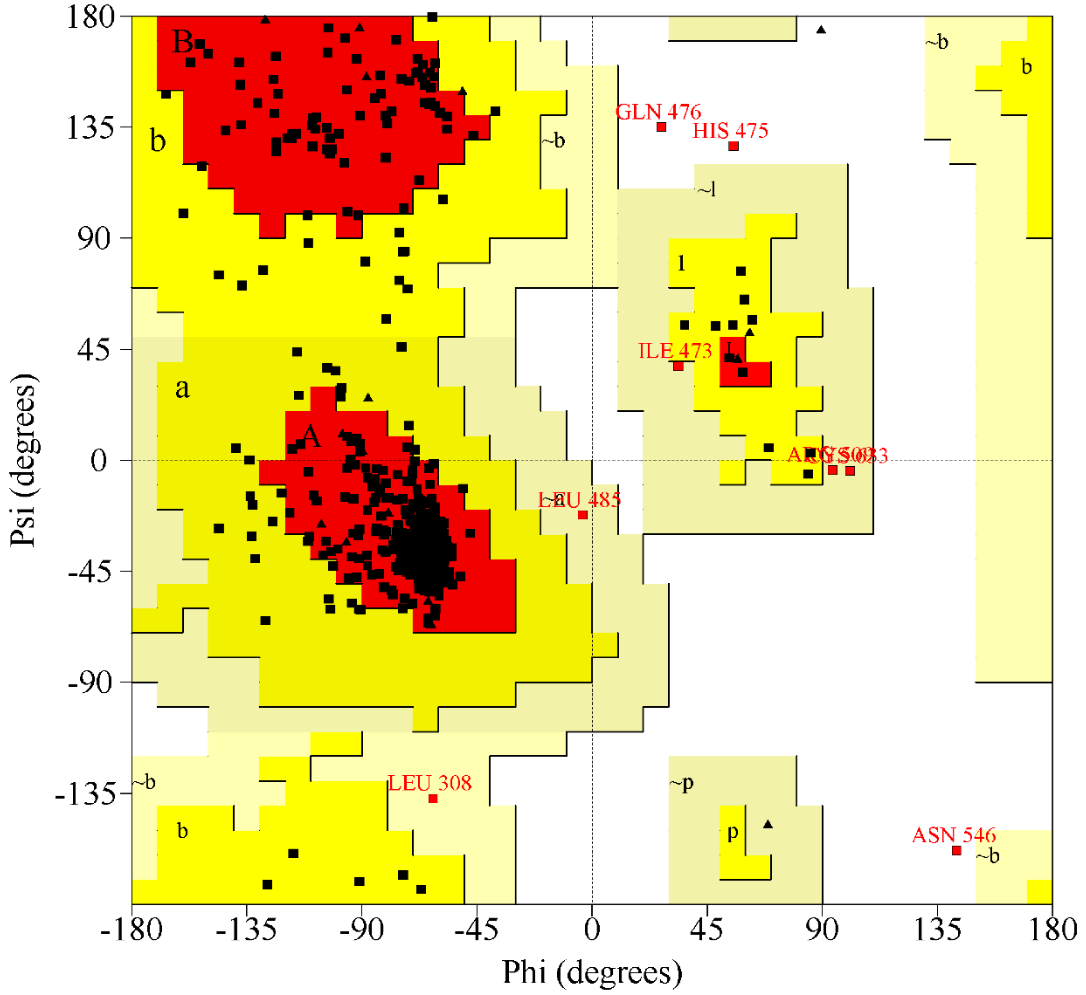


Plot statistics

| | | |
|---|---|---|
| Residues in most favoured regions [A,B,L] | 446 | 87.5% |
| Residues in additional allowed regions [a,b,l,p] | 56 | 11.0% |
| Residues in generously allowed regions [~a,~b,~l,~p] | 5 | 1.0% |
| Residues in disallowed regions | 3 | 0.6% |
| | ---- | ------ |
| Number of non-glycine and non-proline residues | 510 | 100.0% |
| Number of end-residues (excl. Gly and Pro) | 2 | |
| Number of glycine residues (shown as triangles) | 46 | |
| Number of proline residues | 34 | |
| | ---- | |
| Total number of residues | 592 | |

Based on an analysis of 118 structures of resolution of at least 2.0 Angstroms and R-factor no greater than 20%, a good quality model would be expected to have over 90% in the most favoured regions.
